# The Treatment of Periosteal Sarcoma

**Published:** 1908-02-08

**Authors:** 


### SPECIAL ARTICLE.

### THE TREATMENT OF PERIOSTEAL SARCOMA.

If questioned as to the correct treatment for a periosteal sarcoma, the natural impulse of a surgeon is to answer "amputation." And so far as the hypothetical case is concerned this impulse may be right, and the text-books also, from which it originates, may be right. Nevertheless, in actual practice there are at least two reasons for hesitation before recommending such treatment.

### The Fallacy of Uncertain Diagnosis.

In the first place, the practitioner has not a sure diagnosis from which to start out. He has not a hypothetical case of periosteal sarcoma to treat, but a patient who has a swelling which seems to be a periosteal sarcoma. If the surgeon leaps hastily to the conviction that the swelling is without doubt malignant, and from that point strides on with equal inconsequence to perform amputation, he may do irretrievable mischief. Doubtless such an artisan is apt to convey a favourable impression to the lay mind by his assurance and incisive determination; possibly, also, he can find in self-excuse a salve to cure remorse. But his work is not surgery, nor is it to be commended.

Many a limb has been amputated in error, and a supposed sarcoma after amputation has proved to be inflammatory thickening, gumma, callus, or some other innocent formation.

With care some of the sources of error may be removed, but in the early stages at least there is ever the shadow of uncertainty.

A complete examination of the patient is, of course, essential; and the presence of secondary deposits or of multiple lesions especially sought. For if more than one bone be affected, the disease is nearly sure to be inflammatory, because multiple sarcomata of bones are rare, whereas tuberculous and syphilitic inflammations frequently affect more than one bone at the same time. The clinical history, too, needs careful investigation. A short course of anti-syphilitic treatment may be useful to clear up the diagnosis, but it must be vigorous, and not shirked by the patient. Good skiagrams are of the utmost value, and are perhaps the least untrustworthy guides of all, although not infallible. A microscopic section of the tumour is an aid towards certainty, although a laboratory diagnosis is not to be accepted without reserve. Moreover, the practice of cutting a piece out of a malignant tumour for microscopical examination is open to objection, as dissemination of the tumour by this means is believed to be a real danger.

In brief, by means of scrupulous care in examining the patient the chances of error in diagnosis may be diminished, but they cannot be excluded.

### Results of Amputation.

The second reason for hesitation before recommending removal of a limb affected with periosteal sarcoma is that the results of amputation in such a case are apt to be disappointing. Experience has shown that a cure by this means is unlikely; sooner or later secondary sarcomata appear and destroy the patient. It is true that the prognosis after amputation varies to some extent with the position of the primary growth. If the femur or the humerus be the seat of the growth, the hope of saving the patient by amputation is extremely small; in the case of the femur it is so small as to be negligible. With more distally placed growths the prognosis is better, but it is still bad. Furthermore, amputation, especially through the hip-joint or shoulder-joint, is an operation not free from a mortality of its own.

To sum up, it may be stated that a patient with a periosteal sarcoma of his femur is not likely to derive much benefit from an amputation through the hip-joint. If the humerus be affected, life may be prolonged by removal of the limb, but recurrence of the growth is to be anticipated. With periosteal sarcoma of one of the other long bones probably life will be prolonged, and possibly a permanent cure will be obtained.

Incidentally it may be remarked that the majority of all cases occur in connection with the femur or the humerus.

With regard to alternative modes of treatment, Dr. Coley, of New York, has been advocating again the treatment of these cases by injecting the mixed toxins of the streptococcus erysipelatis and the bacillus prodigiosus, a method of which he first made trial some years ago. He now states that in about 10 per cent. of inoperable and apparently hopeless cases injections of the mixed toxins have effected a cure, so that a further and more extended trial of Dr. Coley's treatment may be expedient.

In conclusion, it is to be remembered, in the first place, that there is always an element of doubt in the diagnosis; and, in the second place, that in a large proportion of cases removal of the affected limb is at best but a very poor treatment for periosteal sarcoma.

